# Clinical and Neonatal Outcomes of Children Born After ICSI With or Without Surgically Acquired Sperm: A Retrospective Cohort Study

**DOI:** 10.3389/fendo.2021.788050

**Published:** 2022-01-25

**Authors:** Mingze Du, Junwei Zhang, Zhen Li, Yang Liu, Kexin Wang, Yichun Guan

**Affiliations:** The Reproduction Center, The Third Affiliated Hospital of Zhengzhou University, Zhengzhou, China

**Keywords:** percutaneous epididymal sperm aspiration, testicular sperm aspiration, microdissection testicular sperm extraction, intracytoplasmic sperm injection, live birth rate

## Abstract

**Objective:**

The purpose of this study was to explore the effects of different methods of obtaining sperm for intracytoplasmic sperm injection (ICSI) cycles on the live birth rate (LBR) and neonatal outcomes.

**Methods:**

This was a single-center retrospective cohort study conducted from January 2016 to December 2019. A total of 3557 ICSI cycles were included in the analysis, including 540 cycles in the surgically acquired sperm group and 3017 cycles in the ejaculated sperm group. The main outcome measure was the LBR.

**Results:**

The clinical pregnancy rate in the surgically acquired sperm group was 69.4%, which was significantly higher than the 59.7% clinical pregnancy rate in the ejaculated sperm group (P=0.01). The LBR of the surgically acquired sperm group was significantly higher than that of the ejaculated sperm group (63.1% *vs.* 51.2%, P<0.01). Similarly, the singleton LBR was also higher in the surgically acquired sperm group than in the ejaculated sperm group (45.4% *vs.* 39.2%, P=0.04). Due to differences in the baseline characteristics of the two groups, multiple logistic regression analysis was performed. After multiple logistic regression analysis, the different methods of obtaining sperm were independent risk factors influencing the clinical pregnancy rate (adjusted odds ratio (AOR)=0.73, 95% confidence (CI)=0.56-0.95, P=0.02) and LBR (AOR=0.69, 95% CI=0.54-0.89, P=0.01). The preterm birth rate (AOR=1.42, 95% CI=0.62-3.25, P=0.41) and the incidence of low birth weight (AOR=1.03, 95% CI=0.45-2.34, P=0.95), small for gestational age (AOR=0.81, 95% CI=0.39-1.68, P=0.57), macrosomia (AOR=0.88, 95% CI=0.47-1.66, P=0.70) and large for gestational age (AOR=1.08, 95% CI=0.65-1.82, P=0.76) were not affected by the different methods.

**Conclusion:**

The clinical pregnancy rate and LBR of the surgically acquired sperm group were higher than those of the ejaculated sperm group. There was no significant difference between the neonatal outcomes of the two groups.

## Introduction

According to the results of the latest national reproductive health epidemiological survey conducted in China, the incidence of infertility in China increased from 12% to 18% between 2007 and 2020 (1). Assisted reproductive technology (ART) is considered one of the most effective methods of treating infertility. ART in China has developed rapidly in the past 30 years. The total number of cycles currently exceeds 1 million per year, and the number of babies born exceeds 300,000/year (1). In general, the incidence of infertility caused by male factors is as high as approximately 50% (2, 3). Since the first report in 1992 of the successful application of intracytoplasmic sperm injection (ICSI) technology in the treatment of male infertility and the first live ICSI infant, ICSI has brought hope to many infertile men (4). The effectiveness and safety of this technology have attracted the attention of scholars worldwide. ICSI is based on either ejaculated sperm or surgically acquired sperm; the surgical methods for acquiring sperm include percutaneous epididymal sperm aspiration (PESA), testicular sperm aspiration (TESA) and microdissection testicular sperm extraction (micro-TESE). The reported clinical outcomes based on the different methods of obtaining sperm are not uniform, and few studies have analyzed neonatal outcomes associated with the different sperm acquisition methods (5–7). Therefore, the purpose of this study was to explore the effects of different sperm sources for ICSI on the live birth rate (LBR) and neonatal outcomes to provide evidence for clinical consultation and treatment.

## Materials and Methods

### Population

This was a single-center retrospective cohort study conducted from January 2016 to December 2019 in the reproductive center of the Third Affiliated Hospital of Zhengzhou University. This study was approved by the Ethics Committee of the Third Affiliated Hospital of Zhengzhou University. ICSI cycles with or without surgically acquired sperm were included for potential analysis. Cycles with rescue ICSI, frozen oocyte resuscitation, oocyte donation, age of either spouse ≥ 40 years, basal antral follicle count (AFC) ≤ 3, preimplantation genetic testing (PGT) and incomplete important data were excluded from our study. All basic data, cycle data and offspring follow-up data were obtained from the case system of the reproductive center. All cycles were divided into the surgically acquired sperm group (including groups with sperm acquired *via* PESA, TESA and micro-TESE) and the ejaculated sperm group.

### Clinical Protocols

Mothers underwent our center’s conventional ovulation stimulation protocols, namely, gonadotrophin-releasing hormone (GnRH) agonist protocols or flexible GnRH antagonist protocols. The specific ovulation protocol details have been described in our previous research (8, 9). For both protocols, the dose of follicle-stimulating hormone (rFSH) was adjusted according to follicle response monitoring by transvaginal ultrasound combined with blood hormone levels. As soon as the diameter of the dominant follicle was greater than 20 mm or when at least three follicles reached 18 mm, ovulation induction was triggered with 5000 to 10000 IU human chorionic gonadotrophin (hCG, Lizhu Pharmaceutical Trading, China). Oocyte retrieval was performed 36 hours later.

On the oocyte retrieval day, the male partner prepared semen. (1) For PESA, the patient was instructed to empty his bladder and take a supine position. A sterilized drape was used, and 2% lidocaine spermatic cord block anesthesia was administered with a 5 ml syringe connected to a 4th needle to absorb 1 ml of culture fluid. Percutaneous epididymal puncture was performed with aspiration. Turbid epididymal fluid was observed at the tip of the needle and was transferred to a petri dish. The presence of motile sperm was determined with an inverted microscope. If motile sperm were present, the epididymal fluid was washed and used directly; if no motile sperm were found, the puncture procedure was repeated on the other side of the epididymis. If puncture failed or there was still no motile sperm, TESA was performed for sperm retrieval. (2) For TESA, after emptying the bladder, the patient was placed in a supine position, and disinfection was performed. The patient was administered 2% lidocaine spermatic cord block anesthesia with a 20 ml syringe connected to a 12-gauge needle (with a side hole), and percutaneous testicular puncture was performed. The obtained testicular tissue was injected into a petri dish containing G-MOPS solution (Vitrolife, Switzerland), the tissue was separated and shredded with two 1 ml syringe needles, and the presence of sperm was observed under an inverted microscope. If sperm were detected, the torn tissue suspension was centrifuged at 300 g for 5 min, and the sediment was left in an incubator at 36°C for use. (3) For micro-TESE, after general anesthesia, a sterilized drape was used, and a longitudinal incision was made in the middle of the scrotum. The skin, sarcocarpa, and tunica were incised in turn to expose and squeeze out one testicle and epididymis. Development of the testis and epididymis and the condition of the vas deferens were observed. Under an operating microscope (Carl Zeiss, S88) with the highest magnification of 18 times, a sharp blade was used to cut the albuginea along the transverse plane of the middle of the testis. The testicular tissue was observed, and the testicular parenchyma was carefully removed layer by layer with microtweezers and a microneedle holder. Thick, full, and relatively well-shaped seminiferous tubules were chosen, and the best tubules were removed and immediately sent to the IVF laboratory. These tubules were mechanically shredded by senior embryo laboratory staff, and an inverted microscope (×400) was used to search for sperm. If sperm with good shapes were found, the operation was over; if no sperm were found, other parts of the testis were explored until the entire testicular tissue was searched. If no sperm were found in one testicle, the opposite testicle was cut, and the above method was repeated. The incision of the albuginea was sutured with 5-0 silk sutures. The testis was placed in the sheath, and the incision was closed after continuous suturing. A compression bandage was applied to the scrotum. (4) In the ejaculated sperm group, the patients abstained from sexual intercourse for 3 to 5 days and then masturbated. The semen was processed by density gradient centrifugation. Semen with very rare sperm was washed directly.

After the oocytes were retrieved, the coronal cumulus complex was precultured in fertilization fluid for 1 to 2 hours, the granulosa cells were removed, oocyte maturity was observed, and mature oocytes were subjected to ICSI. Women underwent 1 to 2 cleavage stage embryo transfers on the third day or single fresh blastocyst transfers on the fifth day after fertilization. Routine corpus luteum support, namely, oral dydrogesterone (10 mg twice daily) (Abbott Co. America), was initially provided on the day of oocyte retrieval, and intravaginal administration of 90 mg of a progesterone sustained-release vaginal gel (Merck Co. Germany) was given. Corpus luteum support was performed at least until 55 days after transplantation if pregnancy occurred.

### Outcome Measures and Definitions

The main outcome measure in this study was the LBR, which was defined as at least one live birth after ≥ 28 gestational weeks. The secondary observation indicators were clinical pregnancy [a pregnancy diagnosed by ultrasonographic visualization of one or more gestational sacs or definitive clinical signs of pregnancy; intrauterine pregnancy; and clinically documented ectopic pregnancy (10)], neonatal birth weight, neonatal sex ratio, gestational weeks at delivery, preterm birth (a birth that takes place after 28 weeks and before 37 completed weeks of gestational age), low birth weight (LBW, a neonatal birth weight less than 2500 g), small for gestational age [SGA, a birth weight less than the 10th percentile for gestational age; the weight criteria refer to the weight of Chinese newborns (11)], macrosomia (a neonatal birth weight more than 4000 g), large for gestational age [LGA, a birth weight greater than the 90th percentile of the sex-specific birth weight, with sex-specific birth weight referring to the weight of Chinese newborns (11)] and neonatal congenital malformations.

### Statistical Analysis

All statistical management and analyses were performed using SPSS software version 22.0.

The one-sample Kolmogorov–Smirnov test was used to check for normality of continuous variables. Continuous variables with abnormal distributions are expressed as the median (P25, P75), and the between-group differences were assessed by the Wilcoxon rank sum test. Categorical variables are represented as the number of cases (n) and percentage (%). The means from chi-square analyses were used to assess the differences between groups with Fisher’s exact test when necessary.

Multivariate logistic regression was performed to adjust for potential confounding factors for clinical pregnancy rate, LBR, preterm birth, LBW, SGA, macrosomia and LGA. Adjusted odds ratios (AORs) with 95% confidence intervals (CIs) were calculated. A P value < 0.05 was considered significant.

## Results

### Study Population

Between January 2016 and December 2019, 5871 ICSI cycles with or without PESA/TESA/micro-TESE were performed in the reproductive center of the Third Affiliated Hospital of Zhengzhou University.

**Figure 1:**
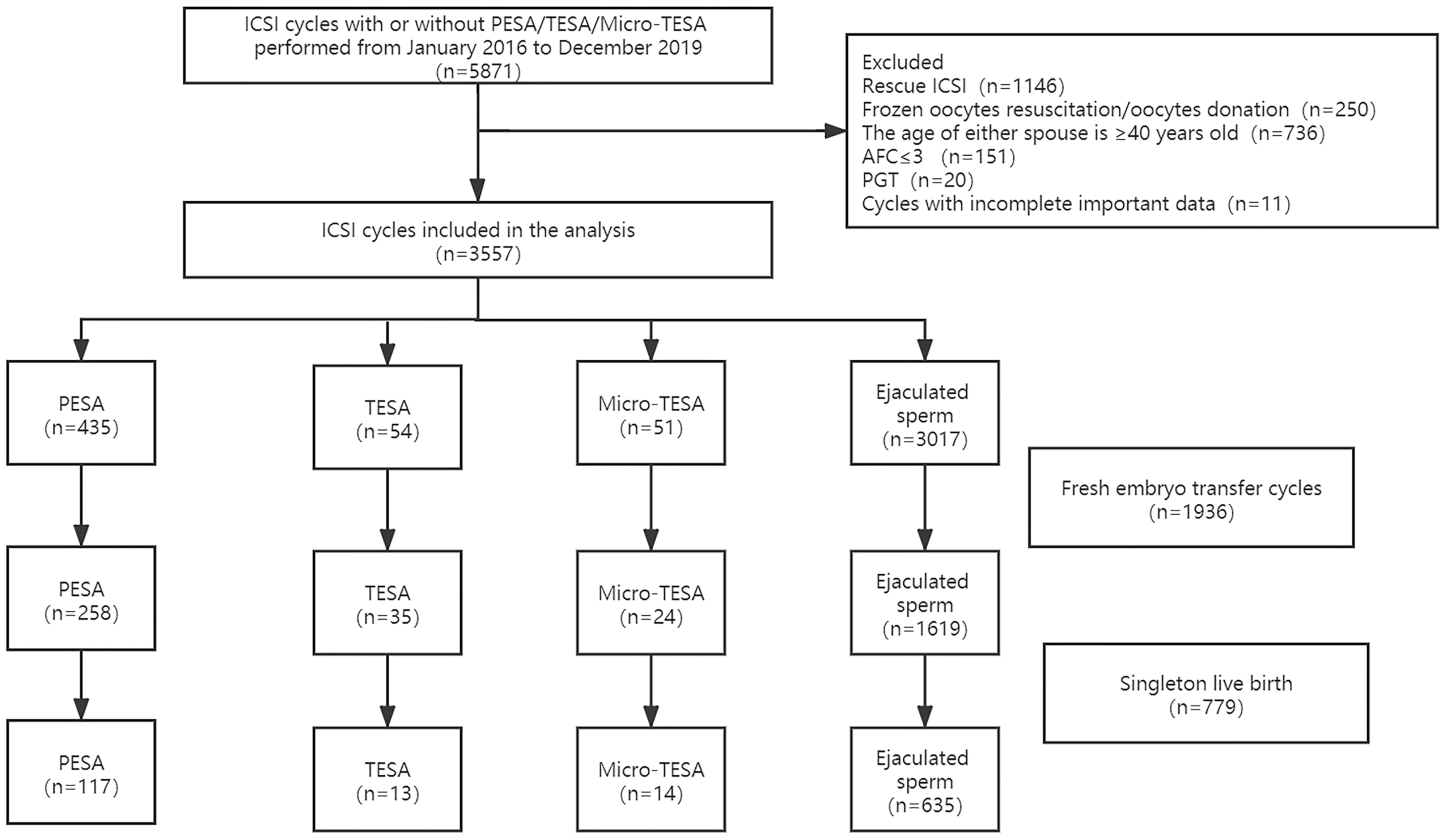
Flow chart of the study design.

After excluding cycles with rescue ICSI (n=1146), frozen oocyte resuscitation/oocyte donation (n=250), age of either spouse ≥ 40 years old (n=736), basal AFC≤ 3 (n=151), preimplantation genetic testing (PGT) (n=20) and incomplete important data (n=11), 3557 ICSI cycles were included in the analysis of this study, namely, 540 cycles in the surgically acquired sperm group (including 435 PESA, 54 TESA and 51 micro-TESE) and 3017 cycles in the ejaculated sperm group. Fresh embryo transfer (ET) was performed in a total of 1936 cycles, and 779 cycles delivered singleton live births. The specific inclusion and exclusion process is shown in **Figure 1**.

### Characteristics of the Study Groups

The detailed baseline and cycle characteristics among the groups are described in **Table 1**. We statistically analyzed and compared the differences between the surgically acquired sperm group and the ejaculated sperm group. There were significant differences in maternal age (P<0.01), paternal age (P<0.01), duration of infertility (P<0.01), maternal basal serum FSH level (P=0.01), paternal serum FSH level (P<0.01), basal AFC, dosage of gonadotropins (P<0.01), endometrial thickness on hCG trigger day (P<0.01), number of oocytes retrieved (P<0.01), number of 2PN (P<0.01), number of available embryos (P<0.01) and embryo stage at transfer (P=0.04). There were no statistically significant differences in maternal BMI (P=0.06), paternal BMI (P=0.08), anti-Müllerian hormone (AMH) (P=0.33), duration of ovarian stimulation (P=0.18) or number of embryos transferred (P=0.46).

**Table 1:** Baseline and cycle characteristics among groups.

	Surgically acquired				Ejaculated sperm	P value (surgically acquired *versus* ejaculated sperm group)
	Total	PESA	TESA	Mirco-TESA		
No. of cycles	540	435	54	51	3017	
Maternal age (year)	28.0 (26.0, 31.0)	28.0 (26.0, 31.0)	29.0 (26.8, 31.3)	29.0 (28.0, 31.0)	30.0 (27.0, 34.0)	<0.01
Paternal age (year)	29.0 (26.0, 32.0)	28.0 (26.0, 31.0)	30.0 (26.8, 33.0)	30.0 (29.0, 32.0)	30.0 (28.0, 34.0)	<0.01
Duration of infertility (year)	2.0 (1.0, 4.0)	2.0 (1.0, 4.0)	2.0 (1.0, 4.0)	3.0 (2.0, 5.0)	3.0 (2.0, 5.0)	<0.01
Maternal BMI (kg/m^2^)	22.6 (20.7, 24.9)	22.6 (20.7, 24.8)	22.1 (19.7, 24.4)	22.5 (21.2, 25.5)	22.9 (20.8, 25.3)	0.06
Paternal BMI (kg/m^2^)	24.8 (22.5, 26.1)	24.8 (22.5,26.0)	24.8 (22.5, 26.2)	25.1 (23.4, 26.6)	25.0 (24.0, 26.2)	0.08
Maternal basal serum FSH level (IU/L)	6.6 (5.4, 8.0)	6.6 (5.4, 8.0)	7.2 (5.4, 8.5)	6.5 (5.6, 7.6)	6.8 (5.6, 8.2)	0.01
Paternal serum FSH level (IU/L)	4.3 (3.1, 6.2)	4.2 (3.0, 5.8)	4.1 (2.7, 6.5)	6.4 (3.8, 26.4)	5.5 (3.7, 8.8)	<0.01
Paternal serum LH level (IU/L)	3.5 (2.5,4.8)	3.4 (2.5,4.4)	3.4 (2.3,4.8)	5.9 (3.4,9.7)	3.8 (2.7,5.4)	0.01
Semen density	0 (0,0)	0 (0,0)	0 (0,0)	0 (0,0)	7.9 (1.2,25.4)	<0.01
progressive	0 (0,0)	0 (0,0)	0 (0,0)	0 (0,0)	16.7 (6.7,32.8)	<0.01
Normal sperm morphology	0 (0,0)	0 (0,0)	0 (0,0)	0 (0,0)	1 (1,3)	<0.01
AMH (ng/ml)	3.0 (1.8, 4.4)	3.0 (1.9, 4.5)	2.4 (1.4, 4.5)	3.2 (1.8, 4.4)	2.8 (1.5, 4.8)	0.33
Basal antral follicle count	17 (12, 22)	17 (13, 22)	13 (10, 22)	19 (12, 24)	15 (10, 22)	<0.01
Duration of ovarian stimulation (days)	13 (11, 14)	13 (11, 14)	13 (11, 14)	13 (11, 14)	12 (11, 14)	0.18
Dosage of gonadotropins (IU)	2187.5 (1640.0, 2925.0)	2100.0 (1625.0, 2750.0)	2312.5 (1481.3, 3075.0)	2475.0 (1925.0, 3275.0)	2400.0 (1800.0, 3075.0)	<0.01
Endometrial thickness on the hCG trigger day (mm)	11.5 (10.0, 13.0)	11.3 (9.8, 13.0)	12.3 (10.9, 13.9)	11.5 (9.4, 13.0)	11.0 (9.0, 12.8)	<0.01
No. of oocytes retrieved	13 (10, 18)	14 (10, 19)	11 (9, 15)	14 (10, 19)	12 (7, 17)	<0.01
No. of 2PN	8 (5, 11)	8 (5, 12)	6 (4, 10)	6 (3, 12)	7 (3, 10)	<0.01
No. of available embryos	6 (3, 9)	6 (3, 9)	5 (3, 8)	5 (2, 8)	5 (2, 8)	<0.01
No. of embryos transferred (%)						0.46
1	30.0 (95/317)	29.1 (75/258)	40.0 (14/35)	25.0 (6/24)	27.9 (452/1619)	
2	70.0 (222/317)	70.9 (183/258)	60.0 (21/35)	75.0 (18/24)	72.1 (1167/1619)	
Embryo stage at transfer (%)						0.04
Cleavage stage	75.4 (239/317)	74.4 (192/258)	74.3 (26/35)	87.5 (21/24)	80.5 (1304/1619)	
Blastocyst stage	24.6 (78/319)	25.6 (66/258)	25.7 (9/35)	12.5 (3/24)	19.5 (315/1619)	

Data are presented as medians (P25, P75) for the continuous variable and %(n/N) for the categorical variable.

### Clinical Outcomes

Fresh ET was performed in a total of 317 surgically acquired sperm cycles and 1619 ejaculated sperm cycles. The clinical pregnancy rate in the surgically acquired sperm group was 69.4%, which was significantly higher than the 59.7% clinical pregnancy rate in the ejaculated sperm group (P=0.01). The LBR of the surgically acquired sperm group was significantly higher than that of the ejaculated sperm group (63.1% *vs.* 51.2%, P<0.01). Similarly, the singleton LBR was also higher in the surgically acquired sperm group than in the ejaculated sperm group (45.4% *vs.* 39.2%, P=0.04). Specific data are described in **Table 2**.

**Table 2:** Clinical outcomes among groups.

	Surgically acquired				Ejaculated sperm	P value (surgically acquired *versus* ejaculated sperm group)
	Total	PESA	TESA	Mirco-TESA		
No. of fresh embryo transfer cycles	317	258	35	24	1619	0.03
Clinical pregnancy rate (%)	69.4 (220/317)	70.2 (181/258)	60.0 (21/35)	75.0 (18/24)	59.7 (967/1619)	0.01
Live birth rate (%)	63.1 (220/317)	63.6 (164/258)	54.3 (19/35)	70.8 (17/24)	51.2 (829/1619)	<0.01
Singleton live birth rate (%)	45.4 (144/317)	45.3 (117/258)	37.1 (13/35)	58.3 (14/24)	39.2 (635/1619)	0.04

Data are presented as %(n/N) for the categorical variable.

### Neonatal Outcomes

Outcome data for singleton newborns are described in **Table 3**. Neonatal birth weight was comparable between the groups (3300.0 (3005.0, 3637.5) *vs.* 3350.0 (3100.0, 3650.0), P=0.20). There were no significant differences in the neonatal sex ratio (P=0.79), gestational weeks at delivery (P=0.28), preterm birth (P=0.31), LBW (P=0.74), SGA (P=0.29), macrosomia (P=0.87), LGA (P=0.70) and neonatal malformation (P=0.72).

**Table 3:** Singleton neonatal outcomes of the groups.

	Surgically acquired				Ejaculated sperm	P value (surgically acquired *versus* ejaculated sperm group)
	Total	PESA	TESA	Mirco-TESA		
No. of cycles	144	117	13	14	635	
Neonatal birth weight (g)	3300.0 (3005.0, 3637.5)	3300.0 (3035.0, 3600.0)	3550.0 (3050.0, 3725.0)	3430.0 (3000.0, 3625.0)	3350.0 (3100.0, 3650.0)	0.20
Neonatal sex (%)						0.79
Male	54.9 (79/144)	53.8 (63/117)	46.2 (6/13)	71.4 (10/14)	56.1 (356/635)	
Female	45.1 (65/144)	46.2 (54/117)	53.8 (7/13)	28.6 (4/14)	43.9 (279/635)	
Gestational weeks at delivery (week)	39 (38, 40)	39 (38, 40)	39 (38, 40)	38 (37, 39)	39 (38, 40)	0.28
Preterm birth (%)	4.9 (7/144)	5.1 (6/117)	0 (0/13)	7.1 (1/14)	7.2 (46/635)	0.31
Low birth weight (%)	5.6 (8/144)	6.0 (7/117)	0 (0/13)	7.1 (1/14)	4.9 (31/635)	0.74
Small for gestational age (%)	7.6 (11/144)	8.5 (10/117)	7.7 (1/13)	0 (0/14)	5.4 (34/635)	0.29
Macrosomia (%)	9.7 (14/144)	11.1 (13/117)	7.7 (1/13)	0 (0/14)	9.3 (59/635)	0.87
Large for gestational age (%)	15.3 (22/144)	14.5 (17/117)	30.8 (4/13)	7.1 (1/14)	16.7 (106/635)	0.70
Neonatal malformation (%)	2.1 (3/144)	1.7 (2/117)	0 (0/13)	7.1 (1/14)	1.6 (10/635)	0.72

Data are presented as medians (P25, P75) for the continuous variable and %(n/N) for the categorical variable.

### Multiple Logistic Regression

For the clinical pregnancy rate, LBR, preterm birth, LBW, SGA, macrosomia and LGA, we performed multiple logistic regression to adjust for the influence of confounding factors. The multiple logistic regression model included maternal age (continuous variable), paternal age (continuous variable), maternal BMI (continuous variable), paternal BMI (continuous variable), duration of infertility (continuous variable), maternal basal serum FSH level (continuous variable), basal AFC (continuous variable), endometrial thickness on the hCG trigger day (continuous variable), number of embryos transferred (1/2), embryo stage at transfer (cleavage/blastocyst) and the method of sperm acquisition (surgically acquired sperm/ejaculated sperm). After multiple logistic regression analysis, the methods of obtaining sperm were independent factors influencing the clinical pregnancy rate (AOR=0.73, 95% CI=0.56-0.95, P=0.02) and LBR (AOR=0.69, 95% CI=0.54-0.89, P=0.01); that is, compared with those in the ejaculated sperm group, the clinical pregnancy rate and LBR were higher in the surgically acquired sperm group. However, the different methods for obtaining sperm did not affect the preterm birth rate (AOR=1.42, 95% CI=0.62-3.25, P=0.41) or the incidence of LBW (AOR=1.03, 95% CI=0.45-2.34, P=0.95), SGA (AOR=0.81, 95% CI=0.39-1.68, P=0.57), macrosomia (AOR=0.88, 95% CI=0.47-1.66, P=0.70) or LGA (AOR=1.08, 95% CI=0.65-1.82, P=0.76). The specific data are described in **Table 4**.

**Table 4:** Adjusted odds ratio of the singleton clinical and neonatal outcomes of the surgically acquired and ejaculated sperm groups.

	Adjusted OR (95% CI)	Adjusted P value
Clinical pregnancy rate	0.73 (0.56-0.95)	0.02
Live birth rate	0.69 (0.54-0.89)	0.01
Preterm birth	1.42 (0.62-3.25)	0.41
Low birth weight	1.03 (0.45-2.34)	0.95
Small for gestational age	0.81 (0.39-1.68)	0.57
Macrosomia	0.88 (0.47-1.66)	0.70
Large for gestational age	1.08 (0.65-1.82)	0.76

Analysis results were adjusted for maternal age, paternal age, maternal BMI, paternal BMI, duration of infertility, maternal basal serum FSH level, basal antral follicle count, endometrial thickness on the hCG trigger day, number of embryos transferred (1/2), and embryo stage at transfer (cleavage/blastocyst). CI, confidence interval.

## Discussion

This single-center large retrospective cohort study included 3557 ICSI cycles. By analyzing the ICSI associated with different semen extraction methods, the patients were divided into a surgically acquired sperm group and an ejaculated sperm group. The results showed that compared with those of the ejaculated sperm group, the clinical pregnancy rate and LBR of the surgically acquired sperm group were higher; in addition, there was no significant difference between neonatal outcomes of the two groups.

### Comparison With Current Studies

Regarding the clinical outcomes associated with the surgically acquired sperm group and ejaculated sperm group, the research conclusions were not consistent with other studies. Tsai CC et al. (12) performed a retrospective study including 126 ICSI cycles performed using TESA from men with azoospermia and 65 ICSI cycles using fresh ejaculated sperm from men with extremely severe oligo-astheno-teratozoospermia. Their results indicated that there was no evidence of differences between the groups in the clinical outcomes and development of the children. A systematic review and meta-analysis performed in 2019 that compared fertility outcomes of TESA and PESA among men with obstructive azoospermia (OA) undergoing ICSI revealed that TESA and PESA yielded similar pregnancy and miscarriage rates for couples receiving ICSI because of OA (13). However, Ben-Ami I et al. (14) revealed that compared with ejaculated sperm cycles, TESA cycles had a significantly higher implantation rate (20.7% *vs.* 5.7%), higher pregnancy rate (42.5% *vs.* 15.1%), and higher LBR (27.5% *vs.* 9.4%) (15). Similarly, in our study, the pregnancy rate and LBR were higher in the surgically acquired sperm group than in the ejaculated sperm group, providing additional evidence of the clinical effectiveness of the surgically acquired sperm methods. For the neonatal outcomes, there was no significant difference between the neonatal outcomes of the surgically acquired sperm and ejaculated sperm groups. Fedder J et al. (6) performed a controlled national cohort study consisting of 466 children born as a result of a surgically acquired sperm method, while the control groups consisted of 8967 (ICSI with ejaculated sperm), 17 592 (IVF) and 63 854 (natural conception) children. Their results showed that children born after a surgically acquired sperm method was used had similar neonatal outcomes, including total malformation rates, as did children born after ICSI and IVF with ejaculated sperm. A retrospective cohort study conducted in 2020 revealed that the live delivery rate per transfer of the surgically acquired sperm group was significantly higher than that of the ejaculated sperm group (45.4% *vs.* 36.7%, P < 0.001). No significantly increased risk of neonatal outcomes of newborns was found in the ICSI treatment of the surgically acquired sperm group (16). Another study involving 530 children born after ICSI with TESA, 194 children born after ICSI with PESA and 2516 ICSI children born using ejaculated sperm revealed that the birth parameters, stillborn rates, preterm birth rates and rates of LBW and very LBW were comparable between the nonejaculated and ejaculated sperm groups (17).

### Possible Biological Mechanism

Over the past 25 years, a large number of studies have also explored the safety of the offspring of ICSI compared with natural pregnancy, including the risks of congenital malformations, epigenetic disorders, chromosomal abnormalities, subfertility, cancer, delayed psychological and neurological development (18–20). However, because subfertility probably influences the risk estimates, it is still difficult to evaluate the safety of ICSI technology. Due to the importance of the ICSI method for the treatment of male factor infertility, further research on its safety is needed.

In this study, compared with those of the ejaculated sperm group, the clinical pregnancy rate and LBR of the surgically acquired sperm group were higher, and the exact biological mechanism was unclear. This may be related to the shorter residence time of sperm in the testis and epididymis than that in the ejaculated sperm group. Sperm exist in the testis and epididymis. The long process of maturation and modification is very strict in time and space. That is, there are differences in the rate of deformity, DNA fragmentation, acrosome integrity, and maturity of sperm obtained by different methods of sperm extraction (21). On the other hand, the differences may also be related to the mechanical damage in the vas deferens caused by the ejaculated sperm. As much as possible, the sperm used in the process of ART should be the most recently produced sperm. The testes and epididymis are the germinal storage locations for sperm. Sperm obtained from an operation are often fresher than ejaculated sperm, and the tissue package is less affected by the outside environment.

It is also believed that even in the process of epididymal puncture, sperm at the proximal end of the epididymis should be used as much as possible, while sperm at the far end are more susceptible to aging, and DNA sperm fragmentation will be higher (22). Therefore, compared with ejaculated sperm, the embryonic development and reproductive outcome of sperm obtained by surgery may be better. There have also been reports that some patients have undergone multiple cycles of ICSI with ejaculated sperm after implantation failure due to poor embryo quality, followed by ICSI with surgically sourced sperm resulting in a successful pregnancy and delivery; therefore, some researchers have proposed that surgically sourced sperm is better than ejaculated sperm for ICSI (15, 23).

### Strength and Limitations

This was a single-center large retrospective cohort study involving 5871 ICSI cycles. This study not only analyzed the LBR of the surgically acquired sperm group and the ejaculated sperm group but also analyzed the safety of surgically acquired sperm regarding the neonatal outcomes in singleton offspring and the incidence of congenital malformation. This study also included micro-TESE, which has been less analyzed in other studies and may provide information for ICSI consultation regarding the use of PESA, TESA or micro-TESE. Of course, our research also has certain limitations. First, this study was a retrospective cohort study. There were differences between the baseline characteristics of the two groups, and there may be confounding factors that affected the outcomes. However, to correct for the influence of confounding factors, we conducted a multiple logistic regression analysis of the observed indicators and set strict inclusion and exclusion criteria. On the other hand, mainly due to the limitation of the amount of data, we did not separately analyze the three methods of obtaining sperm in the surgically acquired sperm group (including PESA, TESA and micro-TESE), which will be analyzed further in future studies.

## Conclusion

In conclusion, the clinical pregnancy rate and LBR of the surgically acquired sperm group were higher than those of the ejaculated sperm group. There was no significant difference between the neonatal outcomes, including neonatal birth weight, neonatal sex ratio, gestational weeks at delivery, preterm birth, LBW, SGA, macrosomia, LGA and neonatal malformation, of the two groups. This study provides additional evidence demonstrating the clinical effectiveness and safety of surgically acquired sperm in terms of neonatal outcomes, and it provides a basis for clinical consultation. The use of surgically obtained sperm in patients with repeated ICSI failure with ejaculated sperm is also worthy of further investigation in prospective randomized controlled studies.

## Data Availability Statement

The raw data supporting the conclusions of this article will be made available by the authors, without undue reservation.

## Ethics Statement

The studies involving human participants were reviewed and approved by Third Affiliated Hospital of Zhengzhou University (2019-18). Written informed consent for participation was not required for this study in accordance with the national legislation and the institutional requirements.

## Author Contributions

MD and JZ selected the population to be included and excluded, completed data statistics and analysis and wrote the manuscript. YG participated in the review and guidance of the research. YL and KW participated in data entry and sorting. ZL participated in the research design, guidance, and manuscript revision. JZ, MD, and ZL contributed equally to this article. All authors contributed to the article and approved the submitted version.

## Funding

This study received financial support from the Henan Provincial Health and Family Planning Commission (Project number: 2018020199).

## Conflict of Interest

The authors declare that the research was conducted in the absence of any commercial or financial relationships that could be construed as a potential conflict of interest.

## Publisher’s Note

All claims expressed in this article are solely those of the authors and do not necessarily represent those of their affiliated organizations, or those of the publisher, the editors and the reviewers. Any product that may be evaluated in this article, or claim that may be made by its manufacturer, is not guaranteed or endorsed by the publisher.
